# High levels of alexithymia in patients with multiple sclerosis

**DOI:** 10.1590/1980-57642018dn12-020015

**Published:** 2018

**Authors:** Audred Cristina Biondo Eboni, Mariana Cardoso, Felipe Moreira Dias, Paulo Diniz da Gama, Sidney Gomes, Marcus Vinicius Magno Goncalves, Suzana Costa Nunes Machado, Adaucto Wanderley da Nobrega, Monica Fiuza Konke Parolin, Sonia Castedo Paz, Heloisa Helena Ruocco, Claudio Scorcine, Fabio Siquineli, Caroline Vieira Spessotto, Carlos Bernardo Tauil, Yara Dadalti Fragoso

**Affiliations:** 1 Department of Neurology, Universidade da Regiao de Joinville, Joinville, SC, Brazil.; 2 Department of Neurology, Universidade Metropolitana de Santos, Santos, SP, Brazil.; 3 Department of Neurology, Hospital de Base do Distrito Federal, Brasilia, DF, Brazil.; 4 Department of Neurology, Pontificia Universidade Catolica de Sao Paulo, campus Sorocaba, Sorocaba, SP, Brazil.; 5 Department of Neurology, Hospital Beneficencia Portuguesa and Hospital Paulistano, Sao Paulo, SP, Brazil.; Hospital Paulistano, Department of Neurology, Sao Paulo, SP, Brazil; 6 Department of Neurology, Hospital de Caridade, Florianopolis, SC, Brazil.; 7 Neurology Clinic, Curitiba, PR, Brazil.; 8 Department of Neurology, Medical School of Jundiai, Jundiai, SP, Brazil.; 9 Department of Neurology, Universidade Regional de Blumenau, Blumenau, SC, Brazil.

**Keywords:** multiple sclerosis, alexithymia, depression, anxiety, psychology, esclerose múltipla, alexitimia, depressão, ansiedade, psicologia

## Abstract

Alexithymia is a personality trait characterized by difficulties identifying and describing feelings. Some researchers describe high levels of alexithymia among patients with multiple sclerosis (MS) but literature data on this subject are scarce.

**Objective:**

The objective of the present study was to characterize findings of alexithymia in patients with MS.

**Methods:**

This cross-sectional case-control study included 180 patients with MS and a matched control group. Data for patients with MS included disease duration, number of demyelinating relapses and degree of neurological disability, as assessed by the Expanded Disability Scale Score (EDSS). In addition, the Hospital Anxiety and Depression (HAD) scale and the Toronto Alexithymia Scale (TAS) were used.

**Results:**

There were 126 women and 54 men in each group, with median age of 37 years and median education of 16 years. Patients with MS had higher degrees of depression (p<0.01), anxiety (p=0.01) and alexithymia (p<0.01) than did control subjects. For individuals with MS, depressive traits (p<0.01), anxious traits (p=0.03), higher age (p=0.02), lower education level (p=0.02), higher degree of disability (p<0.01) and not being actively employed (p=0.03) were associated with higher rates of alexithymia.

**Conclusion:**

Alexithymia was a relevant finding in patients with MS.

## Resumo

Alexitimia é um traço de personalidade caracterizado pelas dificuldades na identificação e descrição dos sentimentos. Alguns pesquisadores descrevem altos índices de alexitimia em pacientes com esclerose múltipla (EM), porém os dados na literatura são escassos.

**Objetivo:**

O objetivo do presente estudo foi caracterizar achados de alexitimia em pacientes com EM.

**Métodos:**

Este estudo transversal incluiu 180 pacientes com EM e um grupo controle pareado. Dados de pacientes com EM incluíram a duração da doença, número de surtos clínicos de desmielinização e grau de incapacidade neurológica avaliados pela Escala Expandida do Grau de Incapacidade (EDSS). Foram também utilizadas a escala Hospitalar de Ansiedade e Depressão (HAD) e a escala de Alexitimia de Toronto (TAS) foram utilizadas.

**Resultados:**

Cada grupo era constituído por 126 mulheres e 54 homens, com mediana de idade de 37 anos e mediana de escolaridade de 16 anos. Pacientes com EM apresentaram maior grau de depressão (p<0.01), ansiedade (p=0.01) e alexitimia (p<0.01) que os controles. Para pessoas com EM, traços depressivos (p<0.01), ansiosos (p=0.03), maior idade (p=0.02), menor nível educacional (p=0.02), maior grau de incapacidade (p<0.01) e o fato de não estar ativamente trabalhando (p=0.03) levaram a maiores níveis de alexitimia.

**Conclusão:**

Alexitimia foi um importante achado em pacientes com EM.

Multiple sclerosis (MS) is an autoimmune, inflammatory and degenerative disease of the central nervous system. It affects mainly young adults and, besides neurological disability (the hallmark of the disease), a variety of psychopathological conditions are described in patients with MS. There are few studies reporting alexithymia rates in patients with MS, and most papers available include around 30-45 patients with or without control subjects. Alexithymia literally means “pushing away emotions” and can be characterized by difficulties identifying and describing feelings, with an externally oriented thinking style. Some define alexithymia as an “affective agnosia”.^1^ Studies on traumatic brain injury, stroke and epilepsy show that these neurological patients have high rates of alexithymia.^2^ It has been proposed that a common nucleus of vulnerable affectivity and difficulty identifying emotions can be found in many patients, suggesting that depression and anxiety may be associated with alexithymia in many cases. The objective of the present study was to assess the levels of alexithymia in patients with MS and compare these with levels found in gender, age and educational level-matched healthy controls. In addition, the clinical parameters of MS and anxiety and/or depressive traits were evaluated in relation to findings of alexithymia.

## Methods

The present study was approved by the Ethics Committee of the Universidade Metropolitana de Santos, SP, Brazil. All participants signed a written consent form prior to enrollment. Patients with relapsing-remitting MS (n=180) regularly attending the outpatient services of the participating institutions were invited to enroll in this study. All patients were being treated in accordance with the recommendations of the Brazilian Ministry of Health protocol for MS. Control subjects (n=180) were drawn from the general population, including individuals accompanying patients to other outpatient clinics, as well as their relatives and friends. Bipolar disease, eating disorders or any other major psychological/psychiatric diagnoses were exclusion criteria for this study.

Demographic data included age, gender and years of education. Clinical data for patients with MS included disease duration, total number of demyelinating relapses and degree of neurological disability, as assessed by the Expanded Disability Scale Score (EDSS).^3^ This scale quantified the level neurological impairment using increments of 0.5 points, with final score ranging from zero (completely normal) to ten (death due to MS). The Hospital Anxiety and Depression (HAD) scale^4^ and the Toronto Alexithymia Scale (TAS)^5^ were used for assessing traits of anxiety, depression and alexithymia in the participants. HAD scores of 8 points or less for either depression or anxiety were considered negative for these conditions, whereas scores of 12 or more points for either depression or anxiety were considered indicative of these conditions. Scores for the TAS can be summarized as no-alexithymia (≤51), alexithymia (≥61) or borderline (52-60).

Statistical analysis was carried out using SPSS 20. The Kolmogorov-Smirnov test was used to confirm whether the data had a normal distribution. For independent samples, Student’s t-test was used for comparison between results. Comparisons among individuals were calculated using one-way ANOVA with the Scheffe’s post-hoc test, while correlations were calculated using Pearson’s coefficient. Fisher´s exact test was used for analyzing categorical data in a contingency table. The magnitude of analyzed effects was assessed by means of linear regression and multivariate analyses. The confidence interval adopted was 95% and the significance level was established as p≤.05.

## Results

There were 126 women and 54 men in each group, with median age of 37 years and median education of 16 years. There were no significant differences in demographic background between patients with MS and control subjects (Table 1). For patients with MS, the median disease duration was eight years (range 1-20 years) and median EDSS was 2.0 (range 0-6.5). A score of 2.0 on the EDSS indicated patients were, on average, fully independent and had minimal disability.

**Table 1:** Alexithymia in multiple sclerosis (MS). Degrees of depression, anxiety and alexithymia in control subjects and patients with multiple sclerosis (MS). Values expressed as mean ± standard deviation.

	Age	Education (years)	Employed	Depressive traits	Anxiety traits	Number of individuals with alexithymia	Number of relapses	Degree of disability
Control subjects (n=180)	39.8±12.2	16.5±4.1	n=157	7.7±3.8	6.0±3.3	n=30		
Magnitude of influence on alexithymia (p value)	p<.01	p<.01	p=.22	p=.40	p<.01			
Patients with MS (n=180)	39.4±11.6	16.0±5.2	n=109	9.2±4.7	7.1±4.1	n=80	5.9±5.1	2.4±1.6
Magnitude of influence on alexithymia (p value)	p=.02	p=.02	p=.03	p<.01	p=.03		p=.33	p=.16
Differences control vs patients	p=.54	p=.28	p<.05	p<.01	p=.01	p<.01		

For individuals with MS, depressive and anxious traits, higher age, lower educational level and not being actively employed had a significant influence on higher rates of alexithymia. For control subjects, higher age, lower educational level and anxiety traits influenced the rates of alexithymia.

Main results are summarized in Table 1. Briefly, patients with MS had higher degrees of depression (p<.01), anxiety (p=.01) and alexithymia (p<.01) than did control subjects. For individuals with MS, depressive traits (p<.01), anxious traits (p=.03), higher age (p=.02), lower educational level (p=.02), higher degree of disability (p<.01) and not being actively employed (p=.03) had a significant influence on higher rates of alexithymia. Control subjects with higher age (p<.01), lower educational level (p<.01) and higher anxiety traits (p<.01) had higher rates of alexithymia, but being actively employed did not affect their results. Mild-to-moderate anxious traits were identified in 13 control subjects and 22 patients with MS, while mild-to-moderate depressive traits were observed in six control subjects and 13 patients. Three patients with MS had severe depression. Alexithymia was identified in 27 controls and 93 patients with MS. High levels of alexithymia were identified in 14.1% of patients with depression (scores ≥12) and 30.8% of patients with anxiety (scores ≥12 on HAD). Regarding control subjects, high levels of alexithymia were identified in 23% of individuals with depression (scores ≥12) and 40% of controls with anxiety (scores ≥12 on HAD). There was no significant difference between (depressive/anxious) patients and controls that had high levels of alexithymia.

Multivariate analyses and linear regression showed that alexithymia was positively associated with anxiety (p=0.0001; beta=0.319) and with depression (p=0.014; beta=0.220) both in control subjects and patients with MS. For the latter, disease duration did not correlate with the presence of alexithymia, but higher levels of neurological disability were associated with the presence of alexithymia (p=0.001; beta=0.240). In patients, there was an association of alexithymia with anxiety, depression and worse neurological disability (r=0.547).

## Discussion

Patients with MS exhibit complex psychopathological traits that may impact their personal and professional lives. Although depression and anxiety in these patients have been widely studied and recognized as important mood disorders that must be addressed, other conditions may also affect the wellbeing of individuals living with MS. Other less-studied conditions may contribute to the complex psychosocial condition of patients with MS, and those caring for them need to be aware that depression and anxiety may only be the tip of the iceberg.

It is interesting to observe that, in alexithymic subjects, the pro-inflammatory and anti-inflammatory cytokine balance may be altered such that a pro-inflammatory profile may predominate.^6^ This association of alexithymia and an inflammatory state seems to be independent of concomitant depression^7^ but may be concurrent with anhedonia.^8^ Considering the balance between pro and anti-inflammatory cytokines is of great importance in the development and progression of MS. Psychological aspects of the disease may, at least in theory, be associated with this cytokine profile. The full picture may be very complex in patients with MS and proper investigation of the psychopathological condition of these patients may lead to a much more satisfactory result from their long-term follow-up. However, it is difficult (if not impossible) to extract some parameters of the final equation. For example, chronic pain, educational level and depression may alter the self-perceived state of health of an individual and influence the presence and degree of alexithymia.^9^^,^ 10

Among the limitations of this study is the lack of investigation on whether psychological findings correlated with particular areas of brain lesions or atrophy. Further studies will consider the correlation of image and psychopathology in MS. The neurophysiological substrate of the impairment in emotional awareness may be found in particular areas of the brain, such as the posterior cingulate cortex^11^ and the frontal lobe.^12^ Cognition must be addressed in detail in further projects, since some studies suggest that cognitive impairment may be associated with low self-perception of health.^13^ In addition, other psychosocial aspects, particularly those related to quality of life, are to be investigated in forthcoming studies by our group.

In conclusion: Alexithymia was a relevant finding in patients with MS and should be addressed when psychological testing and care are considered for these individuals.
